# Associations between Functional Connectivity Dynamics and BOLD Dynamics Are Heterogeneous Across Brain Networks

**DOI:** 10.3389/fnhum.2017.00593

**Published:** 2017-12-07

**Authors:** Zening Fu, Yiheng Tu, Xin Di, Bharat B. Biswal, Vince D. Calhoun, Zhiguo Zhang

**Affiliations:** ^1^School of Biomedical Engineering, Health Science Center, Shenzhen University, Shenzhen, China; ^2^The Mind Research Network, Albuquerque, NM, United States; ^3^Department of Psychiatry, Massachusetts General Hospital and Harvard Medical School, Boston, MA, United States; ^4^Department of Biomedical Engineering, New Jersey Institute of Technology, Newark, NJ, United States; ^5^Guangdong Provincial Key Laboratory of Biomedical Measurements and Ultrasound Imaging, Shenzhen, China

**Keywords:** functional connectivity dynamic, BOLD dynamic, associations between brain dynamics, spatial heterogeneity, resting-state

## Abstract

Functional brain imaging has revealed two types of dynamic patterns of brain in the resting-state: the dynamics of spontaneous brain activities and the dynamics of functional interconnections between spontaneous brain activities. Although these two types of brain dynamics are usually investigated separately in the literature, recent functional magnetic resonance imaging (fMRI) studies have shown that they exhibit similar spatial patterns, suggesting the dynamics of spontaneous brain activities and the dynamics of their interconnections are associated with each other. In this study, we characterized the local blood oxygenation level dependent (BOLD) dynamics and the functional connectivity dynamics (FCD) in the resting-state, and then investigated their between-region associations. Time-varying FC was estimated as time-varying correlation coefficients using a sliding-window method, and the temporal variability of BOLD and time-varying FC were used to quantify the BOLD dynamics and the FCD, respectively. Our results showed that the BOLD dynamics and the FCD exhibit similar spatial patterns, and they are significantly associated across brain regions. Importantly, such associations are opposite for different types of FC (e.g., within-network FCD are negatively correlated with the BOLD dynamics but the between-network FCD are positively correlated with the BOLD dynamics), and they are spatially heterogeneous across brain networks. The identified heterogeneous associations between the BOLD dynamics and the FCD appear to convey related or even distinct information and might underscore the potential mechanism of brain coordination and co-evolution.

## Introduction

The human brain is a highly dynamic system, which is characterized by non-stationary neural activities and represented by ever-changing mental states at time scales ranging from milliseconds to hours (Lehmann and Skrandies, 1980; Lehmann et al., 1987; Allen et al., 2014). Investigating the resting-state brain dynamics from functional magnetic resonance imaging (fMRI) has been an important research theme for its crucial role towards understanding the mechanisms and the relevance of brain’s spontaneous states.

The brain dynamics can be clearly observed from fluctuations of blood oxygen level dependent (BOLD) fMRI signals, which reflect ongoing postsynaptic excitation of local neuronal assemblies. Different brain regions exhibit different degrees of BOLD fluctuations at rest (Zang et al., 2007; Kannurpatti et al., 2008; Biswal et al., 2010), with the default mode network (DMN) showing higher amplitude of low frequency fluctuations (ALFF) in resting-state (Zang et al., 2007; Zou et al., 2008). This observation coincides with the fact that the DMN regions generally have higher neuronal activity (Shulman et al., 1997) and metabolic rate (Raichle et al., 2001) during the resting-state, suggesting an association between the amplitude of fluctuations and the level of local neuronal activity.

Besides the evident dynamics of neural activities, another type of brain dynamics, dynamics of brain connectivity, has also attracted rapidly increasing interests during recent years. Dynamic functional connectivity (FC) indicates the temporal changes of synchronizations between neural activities. Mounting evidence has demonstrated that FC fluctuates significantly within time scales of seconds to minutes during the resting-state (Hutchison et al., 2013; Di et al., 2015), and such FC dynamics (FCD) are related to some physiological and psychological factors. FCD reflect the brain’s evolving network configurations and might be associated with changing patterns of neural communication that subserve certain brain functions. Spatial heterogeneity of FCD has also been reported in the literature: FC between DMN and frontal-parietal network has relatively larger variability, while the within-network FC of visual network, DMN and sensorimotor network (SMN) is more stable than that of orbitofrontal-limbic network (Allen et al., 2014; Zalesky et al., 2014).

Despite great achievements in the research of both types of brain dynamics (BOLD dynamics and FCD), the relationships between them have been seldom explored. Exploring such relationships may provide a better understanding of the characteristics and mechanisms of FCD, because the non-stationarity of signals shall have an influence on the dynamic patterns of FC (Hutchison et al., 2013). In this study, we hypothesized that the two resting-state brain dynamics, local BOLD dynamics (LBD) and FCD, are associated across brain regions. This hypothesis is based on the following two observations. First, brain signal dynamics (Vakorin et al., 2011; Di et al., 2013; Yang et al., 2014; Huang et al., 2016) and FCD (Allen et al., 2014; Zalesky et al., 2014) share similar spatial patterns. For example, some dominant intrinsic connectivity networks (ICNs), such as DMN and frontal-parietal network, do not only exhibit high variability in their local activities (Zuo et al., 2010), but also vary considerably in their connectivity or network metrics (Honey et al., 2007; van den Heuvel and Sporns, 2013; Allen et al., 2014). In contrast, some ICNs with stable activities during the resting-state, such as the SMN, have relatively stable within-network FC (Zalesky et al., 2014). Second, electrophysiological evidence shows that the high-frequency neural activity (such as gamma oscillations) might be closely related to local BOLD signals, while the lower-frequency neural activity (such as theta oscillations) might reflect long-range synchronizations between distinct brain regions. Those high-frequency and low-frequency neural activities have been found to be coupled via both amplitude and phase (i.e., cross-frequency coupling; Allen et al., 2011; Whitman et al., 2013), which further implies potential associations between BOLD activities and their FC.

To validate our hypothesis, we conducted a detailed examination of LBD and FCD across the whole brain and investigated their potential associations using a resting-state fMRI dataset consisting of 102 normal subjects. The LBD were quantified as the temporal variance of BOLD signals at regions of interest (ROIs) defined by the Dosenbach atlas and the FCD were quantified as the temporal variance of the time-varying correlation coefficients between regions. We first showed that two types of dynamics, the LBD and the FCD, exhibit similar spatial patterns across the whole brain. We further correlated the LBD with the FCD across ROIs of the whole brain as well as of each ICN and identified significant across-ROI associations between LBD and FCD. It was found that the LBD are positively correlated with the within-network FCD but negatively correlated with the between-network FCD. Interestingly, the degree of associations shows significant spatial heterogeneity (i.e., different ICNs have largely different degrees of associations between LBD and FCD). In summary, the converging results demonstrated that the BOLD dynamics and the FCD are spatially associated and such associations are heterogeneous among brain networks.

## Materials and Methods

### Data Collection and Preprocessing

We analyzed a publicly available imaging dataset (Oulu) from the 1000 Functional Connectomes Project. After removing data with large head motion (>3 mm or 3°), 102 subjects were included in current study for further analysis (mean age: 21.2 years, range: 20–23 years; 66 females and 36 males). In total 245 resting-state fMRI images were acquired for each subject using a repetition time (TR) of 1.8 s. High-resolution T1 weighted anatomical image for each subject was also available. More information about MRI acquisition of the data could be found at http://fcon_1000.projects.nitrc.org/fcpClassic/FcpTable.html.

The fMRI data were preprocessed using statistical parametric mapping (SPM8^1^) under MATLAB7.6 environment. The images were motion corrected for each subject, and coregistered to the subject’s high-resolution anatomical image. The anatomical images were segmented using the new segment routine in SPM8. Then, the deformation field obtained from segmentation was applied to all functional images to normalize them into the standard MNI space. After that, the functional images were temporally filtered by a second order Butterworth bandpass filter (0.01–0.1 Hz). One-hundred and sixty ROIs were defined using Dosenbach atlas in six different ICNs (Dosenbach et al., 2010): cerebellum network, cingulo-opercular network (CON), DMN, fronto-parietal network (FPN), occipital network, SMN (Detailed MNI coordinates of ROIs are provided in Table A5 of Supplementary Materials). The BOLD response of each ROI was the average of all voxels in this ROI. For each ROI, six rigid body head motion parameters, six head motion derivatives, the first five eigenvectors from white matter (WM) signals, and the first five eigenvectors from cerebrospinal fluid (CSF) signals were regressed out using linear regression. The WM and CSF masks were defined for each subject using the segmented WM and CSF images with a threshold at *p* > 0.99.

### Estimation of Dynamic FC and Definitions of Different Types of FC

For each subject *i* = 1 … *N* (*N* = 102, number of subjects), dynamic FC was estimated with a sliding window method, wherein we computed correlation coefficient matrices corr(*t*)_i_, *t* = 1 … *T* (*T* = 240, number of TRs), from windowed segments. We used a symmetric rectangle window of 30 s and slid in steps of 1 TR. Finally, the dynamic FC estimates, corr(*t*)_i_, were concatenated to form corr_i_, a *C* × *C* ×*T* (*C* = 160, number of ROIs) array representing the changes in correlation between ROIs as a function of time. Here, the window size of 30 s was shown to be a reasonable choice for estimating FCD, because previous studies reported that changes of FC are not sensitive to the window size in the range of 20–40 s (Allen et al., 2014; Li et al., 2014).

Because the FC within one ICN and the FC between different ICNs have largely different dynamic patterns (Zalesky et al., 2014), we further defined two types of FC and investigated them separately: the within-ICN FC was referred to FC between ROIs within the same ICN and the between-ICN FC was referred to as FC between ROIs at different ICNs. Correspondingly, the dynamics of within-ICN FC and the dynamics of between-ICN FC were referred as the within-ICN FCD and the between-ICN FCD.

### Identification of Spatial Heterogeneity of Local BOLD Dynamics and FC Dynamics

We first characterized the LBD and investigated their spatial heterogeneity among ICNs. For each subject, the temporal variance of BOLD signals was calculated for all 160 ROIs to measure the LBD of ROIs. For each ICN, its LBD were calculated as the mean of LBD of all ROIs within this ICN. Nonparametric repeated measures analysis of variance (ANOVA) was used to test the difference of LBD across ICNs. If there exists significant difference, *post hoc* Wilcoxon rank sum test was conducted to examine whether LBD are significantly different between two ICNs.

The FCD was calculated as the temporal variance of Fisher z-transformed time-varying correlation coefficient for each subject. Before analyzing the FCD, we used a statistic to test the FCD (temporal variance of FC) based on the vector autoregressive (VAR) null model (Chang and Glover, 2010; Zalesky et al., 2014). The details of the statistic are provided in Figure S2 of Supplementary Materials. To investigate the spatial heterogeneity of FCD, we conduct statistical analysis only using the FCD that rejects the null hypothesis at two levels: the ICN level and the network-pair level. First, to examine whether FC has similar dynamic patterns with BOLD signals in terms of their spatial distribution, we compared the within-ICN FCD and the between-ICN FCD for each ICN. For one ICN, its within-ICN FCD were calculated as the mean of temporal variance of within-ICN FC between ROIs within this ICN, while its between-ICN FCD were calculated as the mean of temporal variance of between-ICN FC between ROIs of this ICN and ROIs of other ICNs. The Wilcoxon rank sum test was conducted to examine the difference between the within-ICN FCD and the between-ICN FCD for each ICN. Second, to explore more details of the spatial heterogeneity of FCD, we further compared the FCD among 21 network-pairs. The 21 network-pairs were defined as following: 6 within-ICN FC groups and 15 between-ICN FC groups. Similarly, for each subject, the FCD were averaged within each network-pair to obtain the mean FCD of each network-pair. Nonparametric repeated measures ANOVA was used to test the differences of FCD, then Wilcoxon rank sum test was conducted to examine whether the FCD are significantly different among network-pairs. To address the problem of multiple comparisons, the thresholds of significance for above comparisons were corrected by false discovery ratio (FDR; Nichols and Hayasaka, 2003).

### Associations between Local BOLD Dynamics and FC Dynamics

The Pearson correlation coefficient between LBD and FCD was calculated for the exploration of the potential associations between dynamics of local activities and dynamics of their connections. First, for each ROI, its within-ICN FCD were calculated as the mean of the temporal variance of within-ICN FC between this ROI and others ROIs within the same ICN, while its between-ICN FCD were calculated as the mean of the temporal variance of between-ICN FC between this ROI and ROIs of other ICNs. Then we correlated the within-ICN FCD or the between-ICN FCD with the LBD across all ROIs using Pearson correlation coefficient for each subject. The correlation coefficients were Fisher z-transformed and finally, one sample *t*-test was conducted across subjects to examine whether the correlations are significantly different from 0 at the group level.

To explore more details of the associations between brain dynamics, we correlated the within-ICN FCD or the between-ICN FCD with the LBD across ROIs within each ICN. Similarly, one sample *t*-test was conducted across subjects to examine whether the Fisher z-transformed correlations are different from 0 at the group level. Furthermore, to investigate the potential spatial heterogeneity of the associations, the nonparametric repeated measures ANOVA was used to test the difference of correlations among ICNs. After that, two sample *t*-test was conducted across subjects to examine whether the correlations are different between two ICNs at the group level.

## Results

### Spatial Heterogeneity of Local BOLD Dynamics and FC Dynamics across ICNs

**Figure 1:**
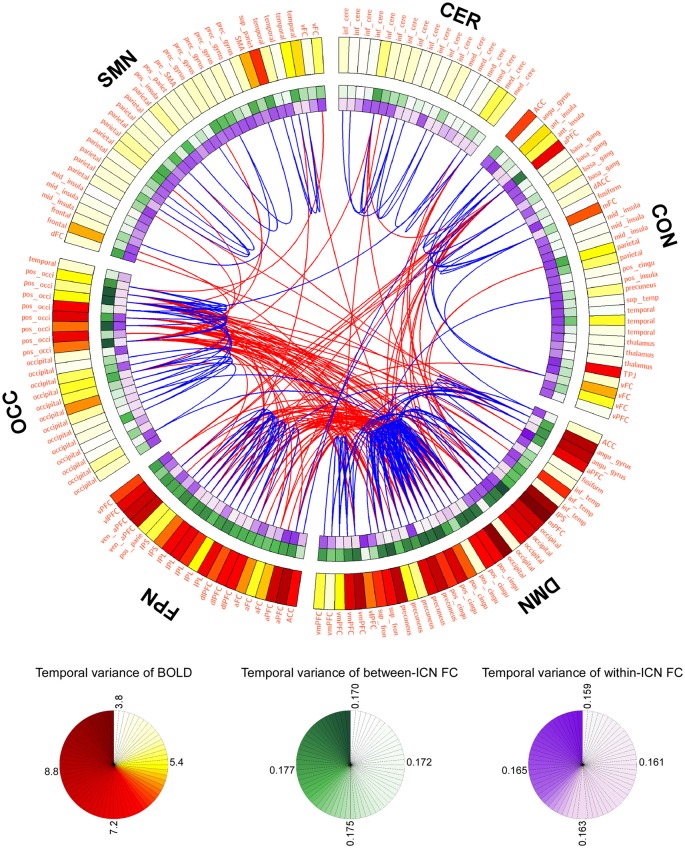
Graphical representation of blood oxygenation level dependent (BOLD) dynamics and FC dynamics (FCD). The connectogram visualizes the temporal variance of BOLD and FC in the whole brain. Background transparency varies such that the most transparent backgrounds are associated with the smallest temporal varaince of BOLD or FC, and the most opaque backgrounds are associated with the largest temporal varaince of BOLD or FC. The most dynamic and statble FC (1%) are displayed in the middle of the connectogram with the red and blue, respectively. Regions of interests (ROIs) were defined using Dosenbach atlas in six different ICNs: CER, cerebellum network; CON, cingulo-opercular network; DMN, default mode network; FPN, fronto-parietal network; OCC, occipital network; SMN, sensorimotor network.

LBD, FCD and the most variable or stable FC are depicted in Figure 1 using a connectogram (Detailed FCD of all FC is provided in Figure S1 of Supplementary Materials). It can be observed from Figure 1 that: (1) BOLD signals within DMN and frontal-parietal network are more variable, and BOLD signals within cerebellum network and CON are relatively stable; (2) the between-ICN FCD within DMN and frontal-parietal network are larger, while the between-ICN FCD within cerebellum network, CON and SMN are relatively smaller; (3) the within-ICN FCD of CON and SMN are larger, while the within-ICN FCD of DMN and frontal-parietal network are relatively smaller; (4) the FC among DMN, frontal-parietal network and occipital network is more variable, while the FC within these ICNs is more stable.

**Figure 2:**
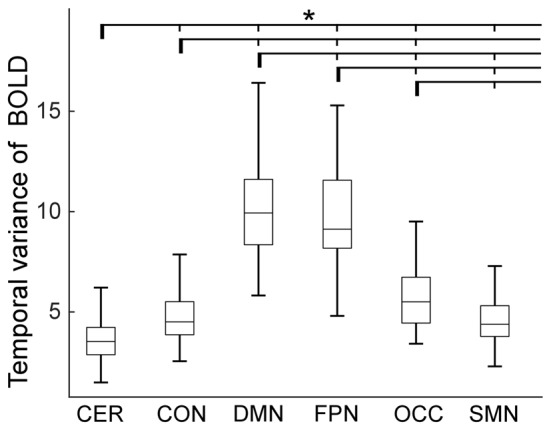
Local BOLD dynamics (LBD) within six ICNs during the resting-state. On each box plot, the central line denotes the median; the edges of the box are the 25th and 75th percentiles. Significant difference between LBD of ICNs is indicated with short thin vertical marks and *(*p* < 0.05, false discovery ratio (FDR) corrected). ROIs were defined using Dosenbach atlas in six different ICNs: CER, cerebellum network; CON, cingulo-opercular network; DMN, default mode network; FPN, fronto-parietal network; OCC, occipital network; SMN, sensorimotor network. The exact *p*-values can be found in Table A1 of the Supplementary Materials.

**Figure 3:**
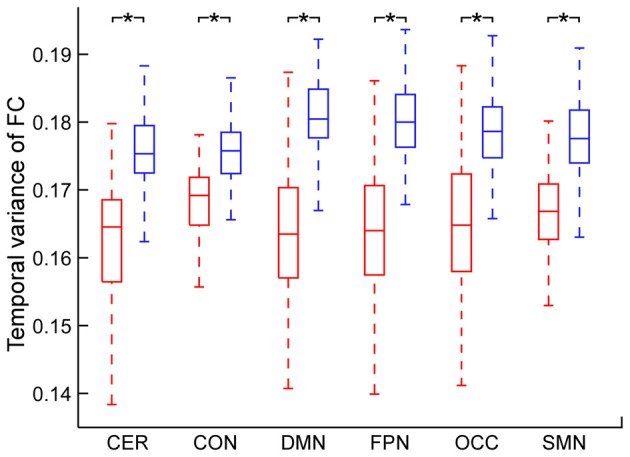
Comparison between within-ICN FCD and between-ICN FCD at each ICN. Within-ICN FCD is marked in red, and between-ICN FCD is marked in blue. On each box plot, the central line denotes the median; the edges of the box are the 25th and 75th percentiles. Significant difference between within-ICN FCD and between-ICN FCD is indicated with *(*p* < 0.05, FDR corrected). ROIs were defined using Dosenbach atlas in six different ICNs: CER, cerebellum network; CON, cingulo-opercular network; DMN, default mode network; FPN, fronto-parietal network; OCC, occipital network; SMN, sensorimotor network. The exact *p*-values can be found in Table A2 of the Supplementary Materials.

**Figure 4:**
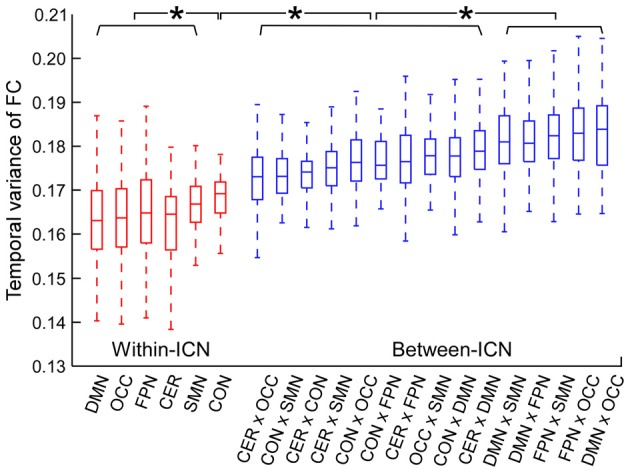
Comparison of FCD between network-pairs. Within-ICN FCD is marked in red, and between-ICN FCD is marked in blue. On each box plot, the central line denotes the median; the edges of the box are the 25th and 75th percentiles. Significant difference between FCD for each pair of ICNs is indicated with *(*p* < 0.05, FDR corrected). ROIs were defined using Dosenbach atlas in six different ICNs: CER, cerebellum network; CON, cingulo-opercular network; DMN, default mode network; FPN, fronto-parietal network; OCC, occipital network; SMN, sensorimotor network. The exact *p*-values can be found in Table A3 of the Supplementary Materials.

The temporal variance of BOLD signals for each ICN is shown in Figure 2. It could be observed that BOLD signals within DMN and frontal-parietal network have significantly larger variability while BOLD signals within cerebellum network, CON and SMN have significantly smaller variability. The comparisons of the temporal variance of FC are shown in Figure 3 (comparison between the within-ICN FCD and the between-ICN FCD for each ICN) and Figure 4 (comparison of the FCD of 21 network-pairs). It can be seen that: (1) the between-ICN FC exhibit significantly larger variability than the within-ICN FC for all ICNs; (2) for the within-ICN FC, the FC within CON and SMN have significantly larger temporal variability than the FC within other ICNs; (3) for the between-ICN FC, the FC between cerebellum network and other ICNs as well as the FC between CON and other ICNs have significantly smaller temporal variability than the other between-ICN FC, while the FC between DMN and other ICNs as well as the FC between frontal-parietal network and other ICNs have significantly larger variability than the other between-ICN FC. These results indicated that BOLD signals and FC have similar dynamic patterns in terms of their spatial distribution (for example, DMN and frontal-parietal network have relative larger LBD and between-ICN FCD, while the other four ICNs have relative smaller LBD and between-ICN FCD).

### Local BOLD Dynamic and FC Dynamics Were Associated

**Figure 5:**
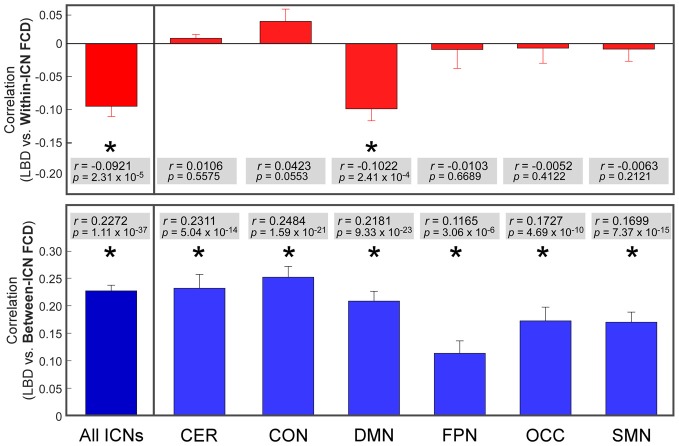
Correlation between LBD and FCD. Significant correlation is indicated with *. ROIs were defined using Dosenbach atlas in six different ICNs: CER, cerebellum network; CON, cingulo-opercular network; DMN, default mode network; FPN, fronto-parietal network; OCC, occipital network; SMN, sensorimotor network.

**Figure 6:**
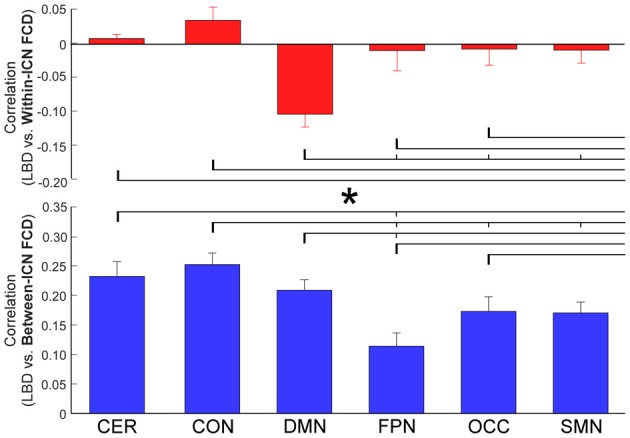
Comparison of correlation between LBD and FCD among six ICNs. Significant difference between correlations of ICNs is indicated with short thin vertical marks and *. ROIs were defined using Dosenbach atlas in six different ICNs: CER, cerebellum network; CON, cingulo-opercular network; DMN, default mode network; FPN, fronto-parietal network; OCC, occipital network; SMN, sensorimotor network. The exact *p*-values can be found in Table A4 of the Supplementary Materials.

The correlation between LBD and within-ICN FCD and the correlation between LBD and between-ICN FCD are depicted in Figure 5. It can be observed a significantly negative correlation between LBD and within-ICN FCD (*r* = −0.0921; *p* = 2.31 × 10^−5^), but a significantly positive correlation between LBD and between-ICN FCD (*r* = 0.2272; *p* = 1.11 × 10^−37^) for the whole resting-state network. The associations between LBD and FCD within each ICN are shown in the right panel of Figure 5. Consistent positive correlations between LBD and between-ICN FCD could still be observed for all 6 ICNs. On the other hand, the negative correlation between LBD and within-ICN FCD is only significant in DMN. We compared the correlations among ICNs and the results are shown in Figure 6. Remarkable spatial heterogeneity of associations between brain dynamics is observed: for the associations between LBD and within-ICN FCD, DMN has the strongest negative correlation among ICNs (although it is near-significant for the comparison with the correlation of cerebellum network); for the associations between LBD and between-ICN FCD, frontal-parietal network has the smallest correlation and CON has significantly larger correlation than frontal-parietal network, occipital network and sensorimotor network have.

## Discussion

In this study, we investigated the spatial characteristics of two types of brain dynamics, the BOLD dynamics and the FCD, and further explored their associations. The main findings were: (1) the BOLD dynamics and the FCD show similar spatial heterogeneity; (2) the FCD are correlated with the BOLD dynamics across brain regions; and (3) the correlations between FCD and BOLD dynamics exhibit spatial heterogeneity.

### Spatial Heterogeneity of Local BOLD Dynamics

The present study explored the temporal variance of BOLD within six ICNs and revealed remarkable spatial heterogeneity of the BOLD dynamics during the resting-state. BOLD activities within DMN were identified to be most variable in our study and this result is consistent with the findings based on ALFF, which is also a mathematical measure of local signal dynamics. Typically, brain regions with greater metabolic and neural activities at rest, such as regions in DMN, have larger ALFF (Zuo et al., 2010). More dynamic activities of DMN might be due to the fact that it is one of the most dominant networks that are recognized as highly active when the brain is at wakeful rest (Raichle et al., 2001). We also speculate that the highly dynamic patterns of some ICNs, such as DMN and FPN might because these ICNs contain some functional hubs of the brain (Hagmann et al., 2008; Tomasi and Volkow, 2011; Smallwood et al., 2012; Liang et al., 2013; Zanto and Gazzaley, 2013). For one thing, the functional hubs are highly connected to the regions within the same ICN. For another, the functional hubs are also functional connected to many regions in other ICNs. Therefore, these hubs and the whole ICNs they belonged to may be more easily to be influenced by the information flow from other brain regions and are therefore highly variable. The high BOLD dynamics in DMN and FPN might also be explained by the dynamic range. The variability of BOLD activity reflects the dynamic range of possible neural responses to incoming stimuli (Garrett et al., 2013). Regions with greater dynamic ranges would permit a greater range of responses to stimulation or tasks. Since DMN and frontal-parietal network participate in various brain functions and are able to respond to a more diversified range of stimuli, a greater dynamic range may result in more variability in their BOLD activity.

### Spatial Heterogeneity of FC Dynamics

Our observation that the within-ICN FC was more stable than the between-ICN FC was consistent with a previous study (Zalesky et al., 2014). Particularly, the FC within DMN, occipital network and frontal-parietal network is most stationary, and in contrast, the FC between these networks is most variable. The spatial heterogeneity of resting-state FCD might be explained by the different information-processing roles of brain regions (Tagliazucchi et al., 2012a,b; Allen et al., 2014; Zalesky et al., 2014). For example, compared with other brain regions, brain regions playing a role in adaptive processes in childhood have relative higher variable FC (Marusak et al., 2017). In the present study, we identified that the within-ICN FC and the between-ICN FC of some dominant networks have significantly different temporal variability (e.g., DMN has the most variable between-ICN FC but the most stable within-network FC). DMN is a coherent system which includes functional hubs of the whole brain network (Tomasi and Volkow, 2011; Smallwood et al., 2012). Our findings implied that these functional hubs also have significantly different temporal variability in their within-ICN FC and their between-ICN FC. Indeed, it could be observed from Figure 1 that some functional hubs (such as PCC and mPFC) have remarkably different within-ICN FCD (more stable) and between-ICN FCD (more variable). These findings could partially explain the inconsistent findings on the dynamics of hub-related FC in previous literature. Shen et al. (2015) showed that hub regions have higher temporal stability in their FC, while some other studies have reported that FC involving hubs is highly variable (Allen et al., 2014). These inconsistent observations on hub-related FC might be due to the different atlases used, which could assign one specific FC to different types (within-network or between-network). The significantly different dynamics of FC might be explained by the following two reasons. For one thing, the within-network communication should be relatively stable to resist the interference from other brain regions so as to maintain their networks’ robustness and adaptability. For another, the between-ICN FC represents the communication between multiple brain sub-systems. These connections typically have relatively longer distances and thus larger workloads (Crossley et al., 2013). To optimize the cost in communication, the between-ICN FC should be maximally variable so that they could switch among hugely possible metastable states.

### Associations between BOLD Dynamics and FC Dynamics

There is scattered evidence showing that the dynamics of brain activities and the connections between brain activities (i.e., static FC) is correlated. For example, larger variability of EEG signals was suggested to reflect the higher integration between distributed neuronal populations (Vakorin et al., 2011). Also, the interaction between temporal variability of BOLD activities and the strength of their synchronization is linearly correlated (Yang et al., 2014). Our previous research also suggested that the strength of FC was influenced by the ALFF in local regions (Di et al., 2013). This study attempts to explore the interaction between brain signal dynamics and FCD (instead of static FC) for a more comprehensive characterization of the temporal complexity of brain dynamics.

We found that LBD and FCD were significantly associated and the associated patterns were different for within-ICN FC and between-ICN FC. One possible explanation of the negative correlation between LBD and within-ICN FCD is that the higher variability in BOLD of one region could enhance the strength and stability of its synchrony with signals at other homogeneous regions. Considering that regions within the same ICN might receive homogeneous input, their within-network connections would be strong and relative stable (Rubinov et al., 2009). Under this situation, noise might be the major cause of the variability in within-ICN FC. For those ICNs with larger activities at rest, such as DMN and frontal-parietal network, the amplitude of neural signal would be larger relative to the noise level (assume that different ICNs might have similar noise levels). When the noise reaches some finite level, the system embedded in an adequate noisy environment (high signal-to-noise ratio, SNR) acquires an enhanced sensitivity towards detection of signals, which is referred to the “stochastic resonance phenomenon” (Gammaitoni et al., 1998; Moss et al., 2004; Rouvas-Nicolis and Nicolis, 2007). As a result, within-ICN FC would be stable, since low-amplitude noise would have less influence on within-ICN FC calculated from high-amplitude signals. In contrast, for those ICNs with less metabolic or neural activity (such as cerebellum network), noises might saturate the neural signals (Garrett et al., 2013). Therefore, within-ICN FC of these ICNs is sensitive to the noise and more variable. An interesting observation is that this negative correlation between LBD and within-ICN FCD could only be identified within DMN, implying this negative correlation may only exist in those “heterogeneous” networks. Among six ICNs, DMN is the heterogeneous network because it is suggested to be comprised of different fractions (Laird et al., 2009; Andrews-Hanna et al., 2010). This may partially explain why the whole resting-state FC network (which is also heterogeneous) and the DMN share similar associations between brain dynamics.

The between-ICN FCD was identified to be positively correlated with the LBD across the whole brain and even within each ICN. One possible interpretation is that the higher variability of local signal could guarantee its between-ICN FC closer to the “critical state” (He, 2011), in which those connections are more flexible and thus more prone to switch among different states. If FC is close to the critical state, it might have high rewiring likelihoods when the brain networks evolve among different reconfigurations. In this situation, noisy dynamics in the brain regions might enable it to persist the ongoing rewiring (Rubinov et al., 2009).

Overall, we argued that the BOLD signal dynamics might modulate FCD in different ways so as to maintain the balance between integration and segregation of brain network, with the purpose of optimizing the cost and efficiency of the brain system. Therefore, this study may contribute to a more complete understanding of the mechanisms of FCD.

### Methodological Considerations and Limitation

First, we used Dosenbach atlas to define 160 ROIs from six ICNs. It should be noted that the ROIs within each ICN were not the same. Therefore, the comparison results might be influenced by the un-balanced ROI size, although the statistical analysis was cautiously used. The dynamic FC in current study was estimated using rectangular windows with a fixed window size. A recent study has shown that tapered windows could provide less sensitive to state transitions and in contrast, rectangular windows could better capture the sharp transition of brain connectivity states (Shakil et al., 2016). However, since it is not clear that brain FC varies sharply or smoothly, we are not sure which types of window could be better used for estimating real dynamic patterns of FC during the resting-state. Researchers also showed that sliding window based FC estimation method would give poor estimates of dynamic FC, if a fixed window size is used. The estimated dynamic FC could not reliably reflect the underlying state transitions unless suitable window sizes (comparable to the state duration) were used. Therefore, adaptive dynamic FC estimation methods with variable window size selection could be used for better capturing the dynamics in the future.

The dynamic patterns and the static patterns of brain might be correlated. Zalesky et al. (2014) have reported evidence showing that a consistent set of FC has significant fluctuations in their connectivity strength over time and the between-ICN (or intermodular) FC is typically more variable than the within-ICN (intramodular) FC. Another study on FCD of neurocognitive networks found similar patterns that the FC between independent components within the same network (or module) is more stable than the FC between independent components in different networks (Marusak et al., 2017). Considering that the within-ICN FC typically has larger connectivity strength than the between-ICN FC has, it is reasonable to assume a negative association between the FC variability and FC strength across the whole brain FC. In current study, although our identified dynamic patterns have similar spatial distribution with the static patterns (e.g., larger variability in FC (between-ICN FC) corresponds to smaller FC strength and smaller variability in FC (within-ICN FC) corresponds to larger FC strength), the detailed relationships between dynamic patterns and static patterns of brain are not explored. Whether and how these patterns are related to each other should be considered and investigated in the future study.

The sliding window approach is the most commonly used method to probe the dynamic patterns in FC. By evaluating two sliding window based methods on an fMRI dataset with extremely large sample size (*n* = 7500), a recent study demonstrated that these two methods can capture highly replicated and reliable dynamic patterns in resting-state FC (Abrol et al., 2017). Abrol et al. (2017) also conducted a surrogate dataset analysis which showed that the dynamic FC patterns captured by the sliding window approaches are indeed statistically significant. Another study used a dataset with simultaneously measured EEG and fMRI data to explore whether the sliding window based approach can capture real dynamic patterns that are corresponding to electrophysiological signatures. They not only identified dynamic FC similar to that observed in previous studies in an independent sample, but also showed that FC changes are highly associated with EEG spectral signatures (Allen et al., 2017). The above findings demonstrated that the sliding window approach is robust against variation in data quality, analysis and grouping, and can capture real variations in FC between brain regions. Although the sliding-window approach has identified numerous valuable dynamic patterns in FC, there are still several critical issues which must be considered in applying the method and interpreting results. Many factors, such as SNR (Hutchison et al., 2013), head motions (Power et al., 2012), variations in the BOLD amplitudes and power (Liu and Duyn, 2013; Fu et al., 2017), and even the approaches (especially the sliding window approach) can induce variations in the estimated FC (Hindriks et al., 2016). A recent study showed that the statistical assessment of results is needed in dynamic FC studies (Hindriks et al., 2016). In the present study, to statistically justify the significance of the observed FCD (temporal variance of FC), we developed a statistic based on the VAR null model. Our results showed that most of the FC rejects the null hypothesis. We evaluate the FC pairs that reject the null hypothesis for further statistical analysis, and observed significant associations between BOLD dynamics and FCD. The converging results suggest that our findings are not caused by sampling variability in the estimation.

## Conclusion

Conventionally, the heterogeneity of brain signals dynamics and brain FCD were investigated separately. Here, we first validated that both dynamics of BOLD and dynamics of FC were different across ICNs and even brain regions. Then we provided evidence showing that dynamics of BOLD were associated with dynamics of FC. Interestingly, similar associations (both positive association and negative association) between brain dynamics were further identified in DMN (for other ICNs, only negative association between LBD and between-ICN FCD could be identified). Overall, our results suggested that there is ubiquitous spatial heterogeneity in dynamics of BOLD, dynamics of FC and their associations. The associations between brain dynamics may provide a new interpretation regarding the evident FCD. Further investigation on brain dynamics and their associations could be performed in various tasks or for different cohorts, which can improve our understanding of a variety of problems (such as brain development, task performance and neurological diseases) related to brain dynamics.

## Ethics Statement

This study used an open-access dataset, and we did not use any identifiable information in our analysis, therefore we did not require IRB approval for the current study.

## Author Contributions

ZZ, VDC and BBB conceived, designed the study and made the critical revision of the article. ZF and XD analyzed the data. ZF and YT wrote the article. All authors discussed the results and implications and commented on the manuscript at all stages.

## Conflict of Interest Statement

The authors declare that the research was conducted in the absence of any commercial or financial relationships that could be construed as a potential conflict of interest.

## Footnotes

^1^http://www.fil.ion.ucl.ac.uk/spm/
