# Supplementary material for: Associations between Functional Connectivity Dynamics and BOLD Dynamics Are Heterogeneous Across Brain Networks
Source: Front Hum Neurosci. 2017 Dec 7;11:593. doi: 10.3389/fnhum.2017.00593 (PMC5770626; doi:10.3389/fnhum.2017.00593)
Supplement: Supplementary file 1 [file Table_1.docx]

Supplementary Materials for

“Associations between Functional Connectivity Dynamics and BOLD Dynamics are Heterogeneous across Brain Networks”

**Zening Fu^1,2,#^, Yiheng Tu^1,3,#^, Xin Di^4^, Bharat B Biswal^4^, Vince Calhoun^2^, Zhiguo Zhang^1*^**

^1^School of Biomedical Engineering, Shenzhen University, Shenzhen, China

^2^The Mind Research Network, Albuquerque, NM, USA

^3^Department of Psychiatry, Massachusetts General Hospital and Harvard Medical School, Boston, MA, USA

^4^Department of Biomedical Engineering, New Jersey Institute of Technology, Newark, New Jersey, USA

**^#^ These authors contribute equally**

* **Corresponding author:**

Dr. Zhiguo Zhang

School of Biomedical Engineering

Health Science Center

Shenzhen University

E-mail: zgzhang@szu.edu.cn

# Statistical results for spatial heterogeneity of local BOLD dynamics and FC dynamics

In the main text, the spatial heterogeneity of local BOLD dynamics (LBD) and FC dynamics (FCD) were investigated. Significant difference in LBD among ICNs (*p* = 2.11×10^-28^) was first identified by the nonparametric repeated measures analysis of variance (ANOVA). Then, the post-hoc Wilcoxon rank sum test was conducted and the detailed results were given in the following Table A1. Significant difference in LBD between ICNs (row < column) was indicated with *, while significant difference in LBD between ICNs (row > column) was indicated with ^#^. To address the problem of multiple comparisons, the significant threshold is set at *p* < 1.31×10^-6^ (FDR corrected threshold for *p* < 0.05). According to the results, the BOLD signals within DMN and FPN had significantly larger variability while those within CER, CON and SMN had significantly smaller variability.

TABLE A1 Statistical Results of the Comparison of Local BOLD Dynamics (*p* values)

| ICNs | CON | DMN | FPN | OCC | SMN |
| --- | --- | --- | --- | --- | --- |
| CER | 2.38×10^-10^ * | 1.22×10^-34^ * | 4.39×10^-34^ * | 4.19×10^-20^ * | 1.20×10^-7^ * |
| CON | - | 2.75×10^-34^ * | 7.37×10^-33^ * | 1.31×10^-6^ * | 0.3027 |
| DMN | - | - | 0.3385 | 4.85×10^-25 #^ | 2.43×10^-32 #^ |
| FPN | - | - | - | 2.44×10^-22 #^ | 5.13×10^-31 #^ |
| OCC | - | - | - | - | 9.15×10^-8 #^ |

We investigated the dynamics of FC in the following two ways. Firstly, we compared within-network FCD (WN-FCD) and between-network FCD (BN-FCD) for each ICN. The detailed statistical results for the Wilcoxon rank sum test were given in the Table A2. Significant difference between WN-FCD and BN-FCD is indicated with *. The significant threshold is set at *p* < 7.89×10^-22^ (FDR corrected for multiple comparison, *p* < 0.05). It could be observed that BN-FC exhibited significantly larger variability than WN-FC for all ICNs. Secondly, to explore more details of the spatial heterogeneity of FCD, we compared FCD among 21 network-pairs. Significant difference in FCD among network-pairs (*p* = 5.14×10^-36^) was firstly identified by the nonparametric repeated measures ANOVA. Wilcoxon rank sum test was conducted to examine whether FCD is significantly different between each two network-pairs and the statistical results were given in the Table A3 (Table A3-1 for CER × CER, CON × CON, DMN × DMN, FPN × FPN, OCC × OCC vs. other network-pairs; Table A3-2 for SMN × SMN, CER × CON, CER × DMN, CER × FPN, CER × OCC vs. other network-pairs; Table A3-3 for CER × SMN, CON × DMN, CON × FPN, CON × OCC, CON × SMN vs. other network-pairs; Table A3-4 for DMN × FPN, DMN × OCC, DMN × SMN, FPN × OCC, FPN × SMN vs. other network-pairs). Significant difference in FCD between network-pairs (row < column) was indicated with *, while significant difference in FCD between network-pairs (row > column) was indicated with ^#^. The significant threshold is set at *p* < 0.0229 (FDR corrected for multiple comparison, *p* < 0.05). The statistical results showed that 1) WN-FC exhibited significantly smaller variability than BN-FC; 2) for WN-FC, FC within CON and SMN had significantly larger temporal variability than FC within other ICNs; 3) for BN-FC, FC between CER and other ICNs as well as FC between CON and other ICNs had significantly smaller temporal variability than other BN-FC, while FC between DMN and other ICNs as well as FC between FPN and other ICNs had significantly larger variability than other BN-FC.

TABLE A2 Statistical Results of the Comparison of FC Dynamics (WN-FC vs. BN-FC, *p* values)

|  | CER | CON | DMN | FPN | OCC | SMN |
| --- | --- | --- | --- | --- | --- | --- |
| WN-FC  vs. BN-FC | 1.22×10^-22^ * | 3.51×10^-23^ * | 6.12×10^-29^ * | 4.52×10^-25^ * | 3.37×10^-27^ * | 7.89×10^-22^ * |

TABLE A3-1 Statistical Results of the Comparison of FC Dynamics (Network-Pairs, *p* values)

|  | CER × CER | CON × CON | DMN × DMN | FPN × FPN | OCC × OCC |
| --- | --- | --- | --- | --- | --- |
| CON × CON | 3.11×10^-9 #^ | - | - | - | - |
| DMN × DMN | 0.4212 | 4.22×10^-8^ * | - | - | - |
| FPN × FPN | 0.6678 | 3.15×10^-4^ * | 0.2214 | - | - |
| OCC × OCC | 0.2151 | 1.32×10^-8^ * | 0.4512 | 0.4166 | - |
| SMN × SMN | 0.2216 | 1.33×10^-5^ * | 2.23×10^-6 #^ | 0.5111 | 1.56×10^-6 #^ |
| CER × CON | 1.25×10^-22 #^ | 1.45×10^-12 #^ | 4.51×10^-18 #^ | 1.52×10^-16 #^ | 9.25×10^-22 #^ |
| CER × DMN | 5.42×10^-24 #^ | 2.32×10^-24 #^ | 6.31×10^-25 #^ | 4.22×10^-24 #^ | 4.52×10^-25 #^ |
| CER × FPN | 2.55×10^-27 #^ | 3.26×10^-12 #^ | 4.56×10^-23 #^ | 5.55×10^-25 #^ | 4.32×10^-24 #^ |
| CER × OCC | 6.12×10^-12 #^ | 3.17×10^-8 #^ | 1.92×10^-16 #^ | 6.77×10^-12 #^ | 5.78×10^-15 #^ |
| CER × SMN | 1.21×10^-24 #^ | 4.87×10^-14 #^ | 4.22×10^-22 #^ | 2.15×10^-19 #^ | 2.99×10^-25 #^ |
| CON × DMN | 7.44×10^-37 #^ | 8.22×10^-25 #^ | 5.69×10^-24 #^ | 8.77×10^-18 #^ | 4.91×10^-22 #^ |
| CON × FPN | 5.72×10^-23 #^ | 6.43×10^-22 #^ | 2.23×10^-24 #^ | 7.22×10^-19 #^ | 4.17×10^-23 #^ |
| CON × OCC | 2.42×10^-24 #^ | 4.52×10^-25 #^ | 6.77×10^-24 #^ | 7.91×10^-25 #^ | 4.29×10^-24 #^ |
| CON × SMN | 5.13×10^-27 #^ | 6.19×10^-13 #^ | 7.26×10^-18 #^ | 5.72×10^-17 #^ | 9.51×10^-16 #^ |
| DMN × FPN | 6.11×10^-32 #^ | 4.53×10^-29 #^ | 6.55×10^-29 #^ | 7.10×10^-25 #^ | 2.13×10^-22 #^ |
| DMN × OCC | 6.16×10^-33 #^ | 4.15×10^-22 #^ | 6.49×10^-28 #^ | 8.91×10^-24 #^ | 5.89×10^-23 #^ |
| DMN × SMN | 4.29×10^-37 #^ | 1.23×10^-29 #^ | 7.29×10^-28 #^ | 8.65×10^-21 #^ | 9.11×10^-28 #^ |
| FPN × OCC | 4.33×10^-34 #^ | 8.42×10^-29 #^ | 2.41×10^-29 #^ | 4.44×10^-22 #^ | 4.52×10^-22 #^ |
| FPN × SMN | 1.50×10^-33 #^ | 6.55×10^-26 #^ | 1.71×10^-27 #^ | 6.78×10^-24 #^ | 4.13×10^-24 #^ |
| OCC × SMN | 8.39×10^-26 #^ | 4.21×10^-26 #^ | 9.29×10^-21 #^ | 7.27×10^-27 #^ | 5.59×10^-22 #^ |

TABLE A3-2 Statistical Results of the Comparison of FC Dynamics (Network-Pairs, *p* values)

|  | SMN × SMN | CER × CON | CER × DMN | CER × FPN | CER × OCC |
| --- | --- | --- | --- | --- | --- |
| CER × CON | 2.22×10^-15 #^ | - | - | - | - |
| CER × DMN | 4.48×10^-25 #^ | 2.41×10^-8 #^ | - | - | - |
| CER × FPN | 5.22×10^-22 #^ | 0.0015 ^#^ | 0.0229 * | - | - |
| CER × OCC | 5.92×10^-11 #^ | 0.1212 | 4.23×10^-8^ * | 1.23×10^-4^ * | - |
| CER × SMN | 8.24×10^-12 #^ | 0.0938 | 5.92×10^-5^ * | 0.0874 | 0.0029 ^#^ |
| CON × DMN | 9.16×10^-26 #^ | 4.11×10^-7 #^ | 0.2091 | 0.1952 | 1.48×10^-8 #^ |
| CON × FPN | 4.27×10^-22 #^ | 2.28×10^-4 #^ | 0.0202 | 0.2048 | 5.24×10^-5 #^ |
| CON × OCC | 9.51×10^-22 #^ | 0.0011 ^#^ | 0.0018 * | 0.7786 | 1.78×10^-6 #^ |
| CON × SMN | 8.78×10^-14 #^ | 0.4819 | 3.21×10^-8^ * | 2.91×10^-4^ * | 0.2161 |
| DMN × FPN | 9.19×10^-24 #^ | 2.15×10^-16 #^ | 0.0091 ^#^ | 2.21×10^-6 #^ | 8.21×10^-15 #^ |
| DMN × OCC | 6.52×10^-22 #^ | 5.24×10^-15 #^ | 0.0022 ^#^ | 4.11×10^-5 #^ | 1.29×10^-12 #^ |
| DMN × SMN | 6.21×10^-35 #^ | 8.65×10^-15 #^ | 0.0409 | 3.33×10^-5 #^ | 4.03×10^-14 #^ |
| FPN × OCC | 4.01×10^-31 #^ | 1.22×10^-15 #^ | 2.55×10^-5 #^ | 9.27×10^-7 #^ | 5.11×10^-15 #^ |
| FPN × SMN | 2.91×10^-26 #^ | 1.79×10^-13 #^ | 0.0301 ^#^ | 5.49×10^-5 #^ | 1.26×10^-13 #^ |
| OCC × SMN | 8.29×10^-23 #^ | 2.25×10^-7 #^ | 0.1112 | 0.2312 | 2.32×10^-9 #^ |

TABLE A3-3 Statistical Results of the Comparison of FC Dynamics (Network-Pairs, *p* value)

|  | CER × SMN | CON × DMN | CON × FPN | CON × OCC | CON × SMN |
| --- | --- | --- | --- | --- | --- |
| CON × DMN | 0.0021 ^#^ | - | - | - | - |
| CON × FPN | 0.0664 | 0.1141 | - | - | - |
| CON × OCC | 0.0910 | 0.2214 | 0.6621 | - | - |
| CON × SMN | 0.0370 | 5.22×10^-8^ * | 4.32×10^-6^ * | 1.09×10^-4^ * | - |
| DMN × FPN | 2.12×10^-18 #^ | 4.78×10^-7 #^ | 5.18×10^-4 #^ | 2.46×10^-8 #^ | 1.92×10^-14 #^ |
| DMN × OCC | 4.55×10^-13 #^ | 3.20×10^-8 #^ | 2.24×10^-7 #^ | 5.12×10^-8 #^ | 1.52×10^-14 #^ |
| DMN × SMN | 2.82×10^-10 #^ | 1.51×10^-5 #^ | 5.19×10^-8 #^ | 5.55×10^-7 #^ | 1.54×10^-15 #^ |
| FPN × OCC | 3.06×10^-12 #^ | 5.26×10^-4 #^ | 4.91×10^-8 #^ | 4.43×10^-9 #^ | 1.67×10^-16 #^ |
| FPN × SMN | 9.17×10^-10 #^ | 4.22×10^-9 #^ | 1.12×10^-4 #^ | 9.27×10^-7 #^ | 3.46×10^-14 #^ |
| OCC × SMN | 0.0011 ^#^ | 0.8911 | 0.2511 | 0.0991 | 1.15×10^-6 #^ |

TABLE A3-4 Statistical Results of the Comparison of FC Dynamics (Network-Pairs, *p* value)

|  | DMN × FPN | DMN × OCC | DMN × SMN | FPN × OCC | FPN × SMN |
| --- | --- | --- | --- | --- | --- |
| DMN × OCC | 0.9121 | - | - | - | - |
| DMN × SMN | 0.2341 | 0.2290 | - | - | - |
| FPN × OCC | 0.4410 | 0.8712 | 0.1251 | - | - |
| FPN × SMN | 0.4569 | 0.4125 | 0.8006 | 0.2223 | - |
| OCC × SMN | 4.16×10^-7^ * | 4.55×10^-7^ * | 4.41×10^-4^ * | 5.91×10^-6^ * | 5.42×10^-4^ * |

# Statistical results for the associations between local BOLD dynamics and FC dynamics

In our current study, we calculated the Pearson correlation coefficient between LBD and FCD to explore the associations between brain dynamics. Such correlations were calculated for the whole resting-state network and also within each ICN. To investigate the potential spatial heterogeneity of the associations, nonparametric repeated measures ANOVA was used to test the difference of correlations. The results showed that correlations are significantly different among ICNs (*p* = 5.43×10^-18^). Pair-wise two sample t-test was then conducted across subjects to examine whether the correlations are different between two ICNs. The detailed results were given in the following Table A4 (Table A4-1 for LBD vs. WN-FCD; Table A4-2 for LBD vs. BN-FCD). Significant difference in correlations between ICNs (row < column) was indicated with *, while significant difference in correlations between ICNs (row > column) was indicated with ^#^. The significant threshold is set at *p* < 0.0023 (FDR corrected for multiple comparison, *p* < 0.05). The results showed that: 1) DMN had relative smaller correlation between LBD and WN-FCD than most of the other ICNs; 2) FPN had the smallest correlation between LBD and BN-FCD among ICNs and CON had relative larger correlation between LBD and BN-FCD than FPN, OCC, and SMN had.

TABLE A4-1 Statistical Results of the Comparison of Correlations between Brain Dynamics

(LBD vs. WN-FCD; *p* values)

| ICNs | CON | DMN | FPN | OCC | SMN |
| --- | --- | --- | --- | --- | --- |
| CER | 0.2141 | 2.33×10^-4 #^ | 0.2111 | 0.4212 | 0.5121 |
| CON | - | 4.22×10^-6 #^ | 0.4421 | 0.8212 | 0.6212 |
| DMN | - | - | 4.87×10^-4^ * | 2.17×10^-4^ * | 5.42×10^-4^ * |
| FPN | - | - | - | 0.5502 | 0.2451 |
| OCC | - | - | - | - | 0.6123 |

TABLE A4-2 Statistical Results of the Comparison of Correlations between Brain Dynamics

(LBD vs. BN-FCD; *p* values)

| ICNs | CON | DMN | FPN | OCC | SMN |
| --- | --- | --- | --- | --- | --- |
| CER | 0.2341 | 0.2215 | 1.15×10^-5 #^ | 0.0586 | 0.0266 |
| CON | - | 0.0531 | 2.51×10^-5 #^ | 0.0023 ^#^ | 9.25×10^-4 #^ |
| DMN | - | - | 0.0009 ^#^ | 0.1261 | 0.1351 |
| FPN | - | - | - | 0.0752 | 0.0252 |
| OCC | - | - | - | - | 0.4471 |

# Coordinates and labels of the defined regions of interesting (ROIs)

TABLE A5-1 MNI Coordinates and Labels of ROIs

| **No** | **x** | **y** | **z** | **Label** | **Network** |
| --- | --- | --- | --- | --- | --- |
| 1 | -34 | -67 | -29 | inf cerebellum | cerebellum |
| 2 | 32 | -61 | -31 | inf cerebellum | cerebellum |
| 3 | -25 | -60 | -34 | inf cerebellum | cerebellum |
| 4 | -37 | -54 | -37 | inf cerebellum | cerebellum |
| 5 | 18 | -81 | -33 | inf cerebellum | cerebellum |
| 6 | -6 | -79 | -33 | inf cerebellum | cerebellum |
| 7 | -21 | -79 | -33 | inf cerebellum | cerebellum |
| 8 | 33 | -73 | -30 | inf cerebellum | cerebellum |
| 9 | -24 | -54 | -21 | lat cerebellum | cerebellum |
| 10 | 21 | -64 | -22 | lat cerebellum | cerebellum |
| 11 | -28 | -44 | -25 | lat cerebellum | cerebellum |
| 12 | -34 | -57 | -24 | lat cerebellum | cerebellum |
| 13 | 14 | -75 | -21 | med cerebellum | cerebellum |
| 14 | 1 | -66 | -24 | med cerebellum | cerebellum |
| 15 | -6 | -60 | -15 | med cerebellum | cerebellum |
| 16 | -16 | -64 | -21 | med cerebellum | cerebellum |
| 17 | 5 | -75 | -11 | med cerebellum | cerebellum |
| 18 | -11 | -72 | -14 | med cerebellum | cerebellum |
| 19 | -2 | 30 | 27 | ACC | cingulo-opercular |
| 20 | -41 | -47 | 29 | angular gyrus | cingulo-opercular |
| 21 | 38 | 21 | -1 | ant insula | cingulo-opercular |
| 22 | -36 | 18 | 2 | ant insula | cingulo-opercular |
| 23 | 27 | 49 | 26 | aPFC | cingulo-opercular |
| 24 | 14 | 6 | 7 | basal ganglia | cingulo-opercular |
| 25 | -20 | 6 | 7 | basal ganglia | cingulo-opercular |

TABLE A5-2 MNI Coordinates and Labels of ROIs

| **No** | **x** | **y** | **z** | **Label** | **Network** |
| --- | --- | --- | --- | --- | --- |
| 26 | -6 | 17 | 34 | basal ganglia | cingulo-opercular |
| 27 | 11 | -24 | 2 | basal ganglia | cingulo-opercular |
| 28 | 9 | 20 | 34 | dACC | cingulo-opercular |
| 29 | 54 | -31 | -18 | fusiform | cingulo-opercular |
| 30 | 0 | 15 | 45 | mFC | cingulo-opercular |
| 31 | 37 | -2 | -3 | mid insula | cingulo-opercular |
| 32 | -30 | -14 | 1 | mid insula | cingulo-opercular |
| 33 | 32 | -12 | 2 | mid insula | cingulo-opercular |
| 34 | -55 | -44 | 30 | parietal | cingulo-opercular |
| 35 | 58 | -41 | 20 | parietal | cingulo-opercular |
| 36 | -4 | -31 | -4 | post cingulate | cingulo-opercular |
| 37 | -30 | -28 | 9 | post insula | cingulo-opercular |
| 38 | 8 | -40 | 50 | precuneus | cingulo-opercular |
| 39 | 42 | -46 | 21 | sup temporal | cingulo-opercular |
| 40 | 43 | -43 | 8 | temporal | cingulo-opercular |
| 41 | -59 | -47 | 11 | temporal | cingulo-opercular |
| 42 | 51 | -30 | 5 | temporal | cingulo-opercular |
| 43 | -12 | -3 | 13 | thalamus | cingulo-opercular |
| 44 | -12 | -12 | 6 | thalamus | cingulo-opercular |
| 45 | 11 | -12 | 6 | thalamus | cingulo-opercular |
| 46 | -52 | -63 | 15 | TPJ | cingulo-opercular |
| 47 | -46 | 10 | 14 | vFC | cingulo-opercular |
| 48 | -48 | 6 | 1 | vFC | cingulo-opercular |
| 49 | 51 | 23 | 8 | vFC | cingulo-opercular |
| 50 | 34 | 32 | 7 | vPFC | cingulo-opercular |
| 51 | 9 | 39 | 20 | ACC | default |
| 52 | -48 | -63 | 35 | angular gyrus | default |
| 53 | 51 | -59 | 34 | angular gyrus | default |
| 54 | -25 | 51 | 27 | aPFC | default |
| 55 | 28 | -37 | -15 | fusiform | default |
| 56 | -59 | -25 | -15 | inf temporal | default |
| 57 | -61 | -41 | -2 | inf temporal | default |
| 58 | 52 | -15 | -13 | inf temporal | default |
| 59 | -36 | -69 | 40 | IPS | default |
| 60 | 0 | 51 | 32 | mPFC | default |
| 61 | 45 | -72 | 29 | occipital | default |
| 62 | -9 | -72 | 41 | occipital | default |
| 63 | -42 | -76 | 26 | occipital | default |
| 64 | -28 | -42 | -11 | occipital | default |
| 65 | -2 | -75 | 32 | occipital | default |
| 66 | 10 | -55 | 17 | post cingulate | default |
| 67 | -11 | -58 | 17 | post cingulate | default |
| 68 | -8 | -41 | 3 | post cingulate | default |
| 69 | 1 | -26 | 31 | post cingulate | default |
| 70 | -5 | -52 | 17 | post cingulate | default |

TABLE A5-3 MNI Coordinates and Labels of ROIs

| **No** | **x** | **y** | **z** | **Label** | **Network** |
| --- | --- | --- | --- | --- | --- |
| 71 | -5 | -43 | 25 | post cingulate | default |
| 72 | 5 | -50 | 33 | precuneus | default |
| 73 | 11 | -68 | 42 | precuneus | default |
| 74 | 9 | -43 | 25 | precuneus | default |
| 75 | -3 | -38 | 45 | precuneus | default |
| 76 | -6 | -56 | 29 | precuneus | default |
| 77 | 23 | 33 | 47 | sup frontal | default |
| 78 | -16 | 29 | 54 | sup frontal | default |
| 79 | 46 | 39 | -15 | vlPFC | default |
| 80 | 6 | 64 | 3 | vmPFC | default |
| 81 | -6 | 50 | -1 | vmPFC | default |
| 82 | 9 | 51 | 16 | vmPFC | default |
| 83 | -11 | 45 | 17 | vmPFC | default |
| 84 | 8 | 42 | -5 | vmPFC | default |
| 85 | -1 | 28 | 40 | ACC | fronto-parietal |
| 86 | 29 | 57 | 18 | aPFC | fronto-parietal |
| 87 | -29 | 57 | 10 | aPFC | fronto-parietal |
| 88 | -42 | 7 | 36 | dFC | fronto-parietal |
| 89 | 40 | 17 | 40 | dFC | fronto-parietal |
| 90 | 44 | 8 | 34 | dFC | fronto-parietal |
| 91 | 40 | 36 | 29 | dlPFC | fronto-parietal |
| 92 | 46 | 28 | 31 | dlPFC | fronto-parietal |
| 93 | -44 | 27 | 33 | dlPFC | fronto-parietal |
| 94 | -48 | -47 | 49 | IPL | fronto-parietal |
| 95 | -41 | -40 | 42 | IPL | fronto-parietal |
| 96 | -53 | -50 | 39 | IPL | fronto-parietal |
| 97 | 44 | -52 | 47 | IPL | fronto-parietal |
| 98 | 54 | -44 | 43 | IPL | fronto-parietal |
| 99 | -32 | -58 | 46 | IPS | fronto-parietal |
| 100 | 32 | -59 | 41 | IPS | fronto-parietal |
| 101 | -35 | -46 | 48 | post parietal | fronto-parietal |
| 102 | 42 | 48 | -3 | vent aPFC | fronto-parietal |
| 103 | -43 | 47 | 2 | vent aPFC | fronto-parietal |
| 104 | 39 | 42 | 16 | vlPFC | fronto-parietal |
| 105 | -52 | 28 | 17 | vPFC | fronto-parietal |
| 106 | -44 | -63 | -7 | occipital | occipital |
| 107 | 17 | -68 | 20 | occipital | occipital |
| 108 | 36 | -60 | -8 | occipital | occipital |
| 109 | -34 | -60 | -5 | occipital | occipital |
| 110 | 39 | -71 | 13 | occipital | occipital |
| 111 | 19 | -66 | -1 | occipital | occipital |
| 112 | -16 | -76 | 33 | occipital | occipital |
| 113 | 9 | -76 | 14 | occipital | occipital |
| 114 | 15 | -77 | 32 | occipital | occipital |
| 115 | 29 | -73 | 29 | occipital | occipital |

TABLE A5-4 MNI Coordinates and Labels of ROIs

| **No** | **x** | **y** | **z** | **Label** | **Network** |
| --- | --- | --- | --- | --- | --- |
| 116 | -29 | -75 | 28 | occipital | occipital |
| 117 | 20 | -78 | -2 | occipital | occipital |
| 118 | -18 | -50 | 1 | occipital | occipital |
| 119 | -29 | -88 | 8 | post occipital | occipital |
| 120 | 13 | -91 | 2 | post occipital | occipital |
| 121 | 27 | -91 | 2 | post occipital | occipital |
| 122 | -4 | -94 | 12 | post occipital | occipital |
| 123 | -5 | -80 | 9 | post occipital | occipital |
| 124 | 29 | -81 | 14 | post occipital | occipital |
| 125 | 33 | -81 | -2 | post occipital | occipital |
| 126 | -37 | -83 | -2 | post occipital | occipital |
| 127 | 46 | -62 | 5 | temporal | occipital |
| 128 | 60 | 8 | 34 | dFC | sensorimotor |
| 129 | 58 | 11 | 14 | frontal | sensorimotor |
| 130 | 53 | -3 | 32 | frontal | sensorimotor |
| 131 | -42 | -3 | 11 | mid insula | sensorimotor |
| 132 | -36 | -12 | 15 | mid insula | sensorimotor |
| 133 | 33 | -12 | 16 | mid insula | sensorimotor |
| 134 | -26 | -8 | 54 | parietal | sensorimotor |
| 135 | -47 | -18 | 50 | parietal | sensorimotor |
| 136 | -38 | -15 | 59 | parietal | sensorimotor |
| 137 | 46 | -20 | 45 | parietal | sensorimotor |
| 138 | -55 | -22 | 38 | parietal | sensorimotor |
| 139 | -38 | -27 | 60 | parietal | sensorimotor |
| 140 | -24 | -30 | 64 | parietal | sensorimotor |
| 141 | 41 | -23 | 55 | parietal | sensorimotor |
| 142 | 18 | -27 | 62 | parietal | sensorimotor |
| 143 | -47 | -12 | 36 | parietal | sensorimotor |
| 144 | 42 | -24 | 17 | post insula | sensorimotor |
| 145 | -41 | -31 | 48 | post parietal | sensorimotor |
| 146 | 10 | 5 | 51 | pre-SMA | sensorimotor |
| 147 | -54 | -22 | 22 | precentral gyrus | sensorimotor |
| 148 | -54 | -9 | 23 | precentral gyrus | sensorimotor |
| 149 | 44 | -11 | 38 | precentral gyrus | sensorimotor |
| 150 | -44 | -6 | 49 | precentral gyrus | sensorimotor |
| 151 | 46 | -8 | 24 | precentral gyrus | sensorimotor |
| 152 | 58 | -3 | 17 | precentral gyrus | sensorimotor |
| 153 | 0 | -1 | 52 | SMA | sensorimotor |
| 154 | 34 | -39 | 65 | sup parietal | sensorimotor |
| 155 | -53 | -37 | 13 | temporal | sensorimotor |
| 156 | -41 | -37 | 16 | temporal | sensorimotor |
| 157 | 59 | -13 | 8 | temporal | sensorimotor |
| 158 | -54 | -22 | 9 | temporal | sensorimotor |
| 159 | 43 | 1 | 12 | vFC | sensorimotor |
| 160 | -55 | 7 | 23 | vFC | sensorimotor |

# Temporal variability of all FC


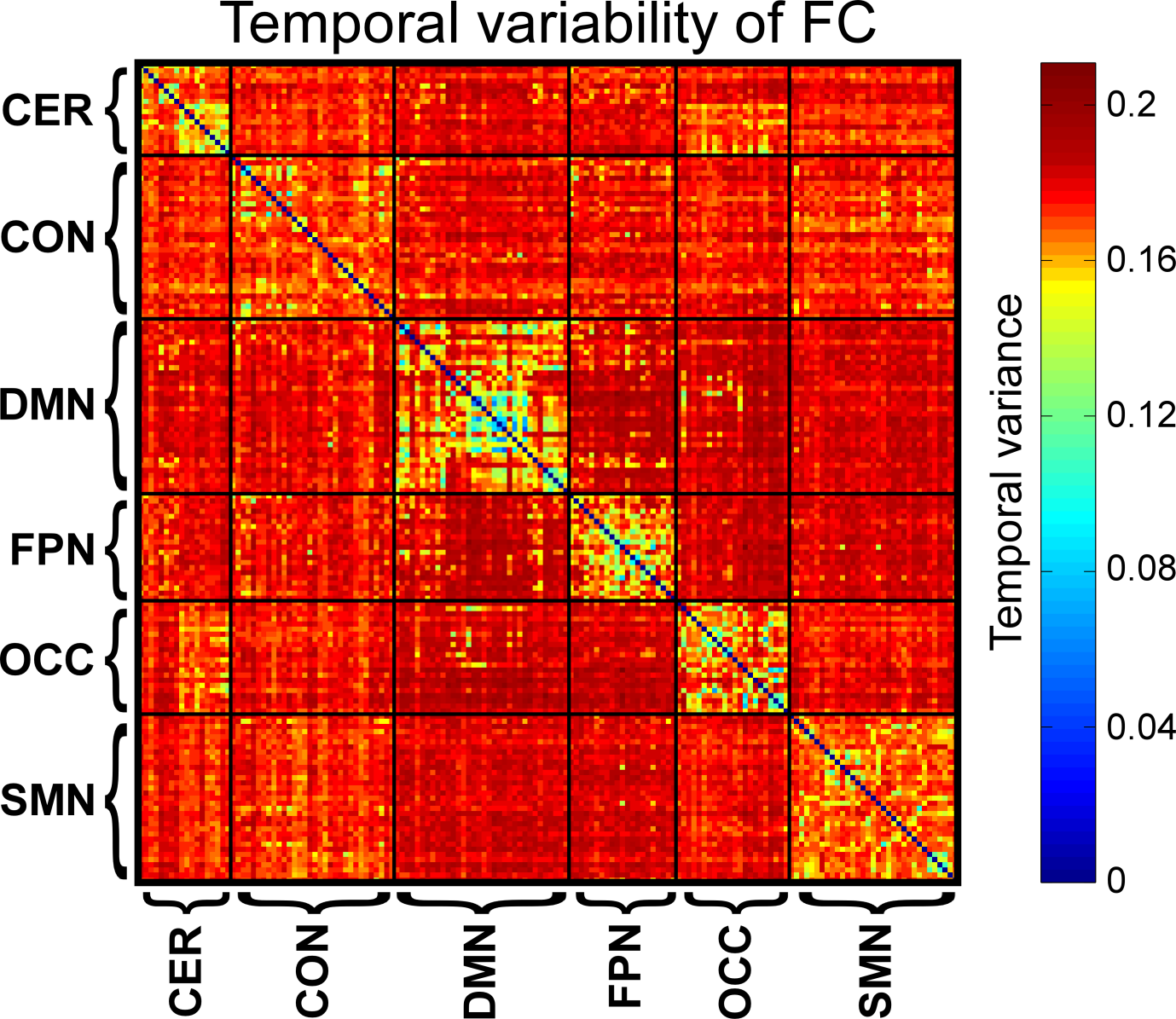


Functional connectivity dynamic (temporal variance) of FC.

# Statistic for testing the FCD

We have conducted a statistic testing for the FCD to show that our identified FCD (temporal variance of FC) is not caused by the sampling variability in the estimation. We developed a statistic to test for FCD (temporal variance of FC) based on the Vector Autoregressive (VAR) Null Model ([Chang et al., 2010](#_ENREF_5); [Zalesky et al., 2014](#_ENREF_29)). Since in the current study we used a fMRI dataset with relative larger sample size (102 subjects, compared with 10 subjects in ([Zalesky et al., 2014](#_ENREF_29)) and 12 subjects in ([Chang et al., 2010](#_ENREF_5))), our statistic is a little bit different from previous studies using VAR and focuses on testing the significance at the group level.

Because it was computationally infeasible to fit a single multidimensional VAR model with a covariance structure with dimensions equal to the number of ROIs ([Chang et al., 2010](#_ENREF_5); [Zalesky et al., 2014](#_ENREF_29)), two-dimensional VAR were independently fitted to each pair of regional time-series. The VAR model order was chosen using the Bayesian information criterion (BIC). The BIC was evaluated for model orders between 1 and 20 for 1000 pairs of regions randomly sampled from all subjects. The optimal model order is 5 (with maximum lag of approximately 9 seconds), and this selection was consistent with a previous study ([Zalesky et al., 2014](#_ENREF_29)). For each pair regions, 200 bootstrap time-series pairs that approximately preserved the power and cross-spectrum of real data were generated using VAR models. Each bootstrap time series pair was generated as follows

1. Select a subject index sub_idxˆ (1 ≤ sub_idx ≤ 102) uniformly at random.
2. Estimate AR coefficients least-squares fit of the *p*th-order VAR model to the original data.
3. Simulate VAR model responses using an approach previously in ([Chang et al., 2010](#_ENREF_5)).

After obtaining the 200 bootstrap time-series pairs, we then used the sliding window approach with the same window size to estimate dynamic FC between these time-series and calculated the FCD (temporal variance of FC) for these simulated data. A two-sample t-test was conducted to examine whether the FCD of simulated data and the FCD of real data is different at the group level. The null hypothesis was rejected pair-wise when the FCD of real data is significantly higher than the FCD of simulated data. To address the problem of multiple comparisons, the thresholds of significance for above comparisons were corrected by FDR with q = 0.05. The results showed that most of the FC rejects the null hypothesis, except for some within-ICN FC, especially within the DMN and FPN.


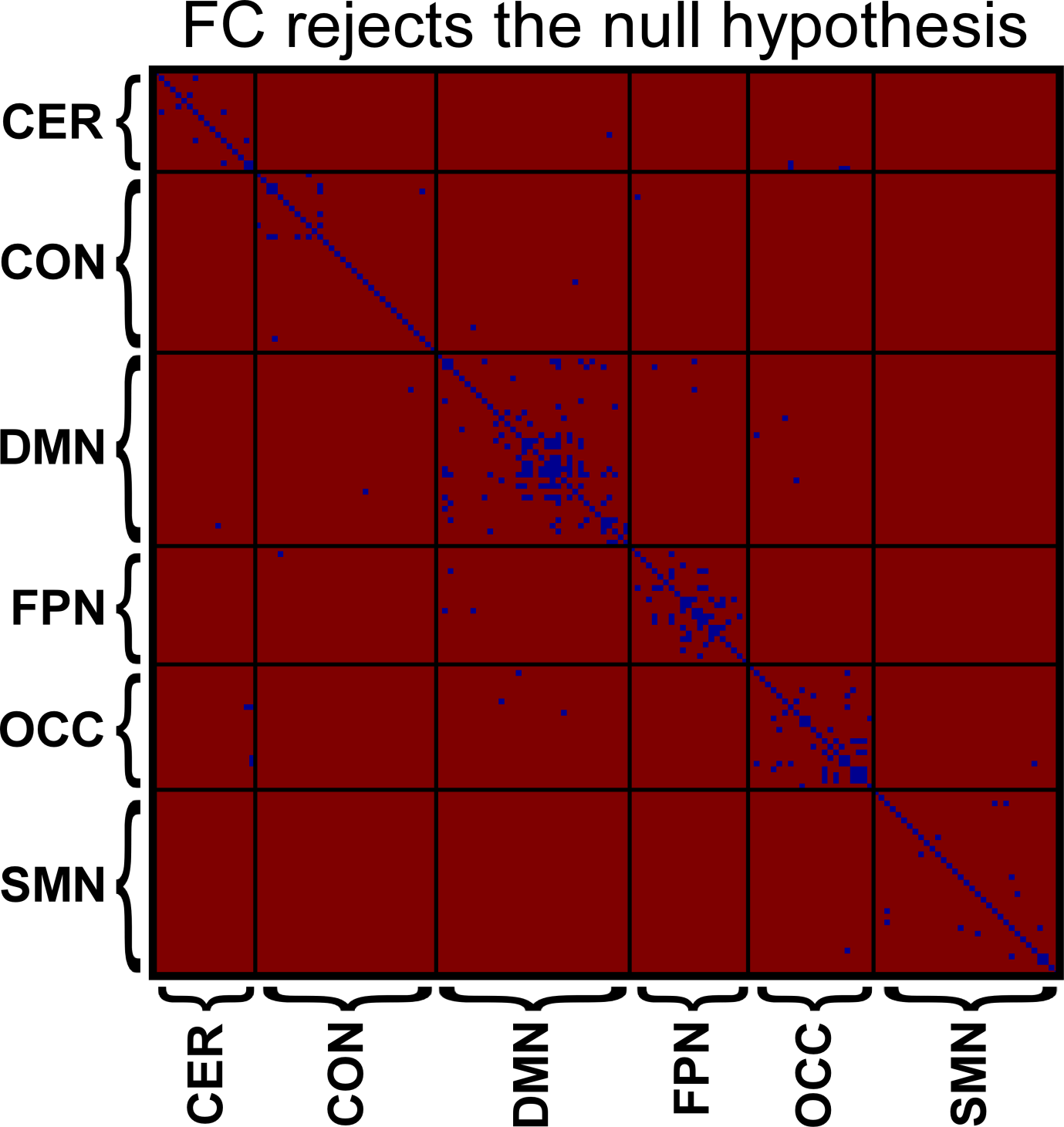


FC rejects the null hypothesis (marked on red, p < 0.05, FDR corrected)
